# Caesarean section epidemic: Tackling the rise of unnecessary cuts

**DOI:** 10.18332/ejm/105892

**Published:** 2019-03-26

**Authors:** Renata Josi

**Affiliations:** 1 University of Applied Sciences and Arts of Southern Switzerland (SUPSI), Department of Business Economics, Health and Social Care, Manno, Switzerland

**Keywords:** physiology, ceasarean section, midwife-led care, unnecessary ceasarean section

**Dear Editor**,

The caesarean section (CS) has a long history in human culture. Once it was only carried out on dying or an already dead woman in the desperate hope to save the child (or being able to bury the dead child apart from the mother). Today the rate of childbirths through CS is consistently rising in high- and middle-income countries. On the other hand, there are still countries in the world where access to a safe CS is not guaranteed. This imbalance and the sharply increasing CS rates do not seem to be justifiable in light of the WHO recommendations, which in 2015 stated that CS rates of more than 10% were not associated with lower maternal or newborn mortality^1^. When looking at the actual CS rates throughout the world, the problem becomes even more accentuated.

Globally, 21.1% of all livebirths in 2015 were estimated to be by CS. From 2000 to 2015 this rate increased from 16 million to nearly 30 million livebirths. However, the rate of CS varies considerably between world regions, from 4.1% in Africa to 44.3% in the Latin America and the Caribbean^2^. But what is even more striking are the differences in CS rates within countries. In 62 low- and middle-income countries, the CS rates in the richest five were at least twice as high as in the poorest five^3^. Differences also occur regionally, as exemplified by Ethiopia where the national average CS rate was 2%, but Addis Ababa (its capital city) reported a CS rate of 21.4%. Furthermore, China and India report great differences in CS rates within their countries (4%–62% and 7%–49%, respectively). This phenomenon is also evident across Europe and the United States where differences up to a factor of two in the CS rate exist between health institutions^2^. In addition to regional differences, the type of service provision has a considerable impact on CS rates. Studies from Australia, India and Brazil show that CS rates are higher in private hospitals than in public hospitals^4-6^. This difference is very impressive in India where a big cross-sectional study of nearly 25000 births showed that the CS rates varied from 13.7% in public hospitals to 37.9% in private hospitals^4^. When looking at these numbers, it is understandable that the increasing CS rates are perceived as an epidemic. So where does this rise come from?

It is without question that CS, when medically indicated, can save lives of mothers and babies and that an access to safe CS should be guaranteed where it is needed but underused. Nevertheless, it has also been shown that CS can do harm to mothers and babies when overused without medical indication. There are many individual, societal and organizational drivers of the overuse of CS. Individual factors such as fear of pain, cultural beliefs or perceived increased safety for mother and child as well as the increase in the access to media and information sources have influenced a woman’s choice^3,7^. Furthermore, women today are older when having their first child, which has led to more complex care needs during childbirth due to an increased potential for more chronic diseases and psychological disorders to arise. In addition, medicalization of the birth process and financial incentives have had an influence on CS rates^3,7^. Finally, the sharp increase in CS rates seems to be also attributable to many unnecessary (without medical indication) caesarean sections. To tackle this issue WHO has recently published its first recommendations to reduce unnecessary caesarean sections.

WHO proposed a collaborative midwife-obstetrician model of care where care is primarily provided by midwifes with a 24-hour back-up from an experienced obstetrician^1^. However, the evidence base for such models to reduce unnecessary caesarean sections is still weak. To safeguard high quality childbirth across the world, the focus from a disease-oriented system where childbirth is seen as a potential risk for mother and child, has to be shifted back to a culture where childbirth is again seen as a natural process. To support this approach to childbirth care, midwives are certainly the best-qualified professional group to take care of women before, during and after childbirth. They have the knowledge and the skills and most importantly, they see and treat childbirth as a naturally occurring process in the lifetime of a woman. It has been shown in a Cochrane Review that women who are cared for by midwifes experience less regional analgesia and are more likely to give birth naturally^8^. There are many other studies that show that midwife-led care is for the best for women who are having a child^9,10^. To solve the problems related to the epidemic of CS, we need the involvement of all stakeholders such as hospitals, insurers, health system administrators, regulators, providers, and the mothers themselves. To make the step towards a physiology-oriented model where midwives care one-to-one for a woman giving birth, a good evidence base as well as support from health policy makers and payers are needed. If childbirth is seen again as a natural process, where CS is done only when necessary, this will result in safer, more satisfying and less expensive care for all women expecting a child.
